# Supplementary material for: Simultaneous detection and quantification of multiple pathogen targets in wastewater
Source: medRxiv. 2023 Dec 5:2023.06.23.23291792. Originally published 2023 Jun 29. Preprint. [Version 2] doi: 10.1101/2023.06.23.23291792 (PMC10327253; doi:10.1101/2023.06.23.23291792)
Supplement: Supplement 12 [file media-12.docx]

References

1. Liu J, Gratz J, Amour C, Nshama R, Walongo T, Maro A, et al. Optimization of Quantitative PCR Methods for Enteropathogen Detection. Chan KH, editor. PLoS ONE. 2016;11: e0158199. doi:10.1371/journal.pone.0158199

2. Rudko SP, Ruecker NJ, Ashbolt NJ, Neumann NF, Hanington PC. Enterobius vermicularis as a Novel Surrogate for the Presence of Helminth Ova in Tertiary Wastewater Treatment Plants. Dudley EG, editor. Appl Environ Microbiol. 2017;83: e00547-17. doi:10.1128/AEM.00547-17

3. Kann S, Hartmann M, Alker J, Hansen J, Dib JC, Aristizabal A, et al. Seasonal Patterns of Enteric Pathogens in Colombian Indigenous People—A More Pronounced Effect on Bacteria Than on Parasites. Pathogens. 2022;11: 214. doi:10.3390/pathogens11020214

4. Sow D, Parola P, Sylla K, Ndiaye JL, Delaunay P, Halfon P, et al. Performance of Real-Time Polymerase Chain Reaction Assays for the Detection of 20 Gastrointestinal Parasites in Clinical Samples from Senegal. The American Journal of Tropical Medicine and Hygiene. 2017;97: 173–182. doi:10.4269/ajtmh.16-0781

5. Qvarnstrom Y, Visvesvara GS, Sriram R, da Silva AJ. Multiplex Real-Time PCR Assay for Simultaneous Detection of *Acanthamoeba* spp., *Balamuthia mandrillaris* , and *Naegleria fowleri*. J Clin Microbiol. 2006;44: 3589–3595. doi:10.1128/JCM.00875-06

6. Costafreda MI, Bosch A, Pintó RM. Development, Evaluation, and Standardization of a Real-Time TaqMan Reverse Transcription-PCR Assay for Quantification of Hepatitis A Virus in Clinical and Shellfish Samples. Appl Environ Microbiol. 2006;72: 3846–3855. doi:10.1128/AEM.02660-05

7. Lu X, Wang L, Sakthivel SK, Whitaker B, Murray J, Kamili S, et al. US CDC Real-Time Reverse Transcription PCR Panel for Detection of Severe Acute Respiratory Syndrome Coronavirus 2. Emerg Infect Dis. 2020;26: 1654–1665. doi:10.3201/eid2608.201246

8. Stokdyk JP, Firnstahl AD, Spencer SK, Burch TR, Borchardt MA. Determining the 95% limit of detection for waterborne pathogen analyses from primary concentration to qPCR. Water Research. 2016;96: 105–113. doi:10.1016/j.watres.2016.03.026

List of Legends

**S1 Table.** pH, temperature and Total Suspended Solids (TSS) for all wastewater influent (n=30)

**S2 Table. Matched samples comparison on TAC for pathogen types**

**S3 Table.** qPCR Primer and Probe Sequences for TAC

**S4 Table.** MIQE Checklist

**S5 Table.** dPCR Primer and Probe Sequences

**S6 Table.** Prevalence of pathogens detected in wastewater influent from four treatment plants in Atlanta, Georgia – using InnovaPrep concentrating pipette pellet

**S7 Table.** dMIQE Checklist

**S8 Table.** BCoV percent recovery by sample

**S9 Table.** Mean log_10_ gene copy concentrations per liter of WW influent (standard deviation) before normalization, by WW Treatment plant – skim milk flocculation

**S10 Table.** Normalization using mtDNA – mean (standard deviation) - excluding duplicates

**S11 Table.** Normalization using PMMoV— mean (standard deviation) - excluding duplicates

**S12 Table.** TAC performance and 95% LOD

**S13 Table.** 95% Matrix Limit of Detection (gene copies per mL sewage)

**S1 Text.** Multiplex assay optimization (BCoV, PMMoV, N1, mtDNA)

**S2 Text.** dPCR assay details, including positive and negative control results

**S1 Fig.** BCoV, PMMoV, mtDNA dPCR RFU plots displaying threshold partitioning for samples, positive and no template controls (NTC)

**S2 Fig.** InnovaPrep Concentrating Pipette Pellet TAC Results

**S3 Fig.** Direct extraction TAC boxplot

**S4 Fig.** Skim Milk Flocculation TAC Boxplot Results by gene target

**S5 Fig.** Amplification and multicomponent plot for no-template control. The amplification occurs for MS2, PhHV, manufacture internal control, and 16S.
