# Supplementary material for: Simultaneous detection and quantification of multiple pathogen targets in wastewater
Source: medRxiv. 2023 Dec 5:2023.06.23.23291792. Originally published 2023 Jun 29. Preprint. [Version 2] doi: 10.1101/2023.06.23.23291792 (PMC10327253; doi:10.1101/2023.06.23.23291792)

**S5 Fig.** Amplification and multicomponent plot for no-template control. The amplification occurs for MS2, PhHV, manufacture internal control, and 16S.


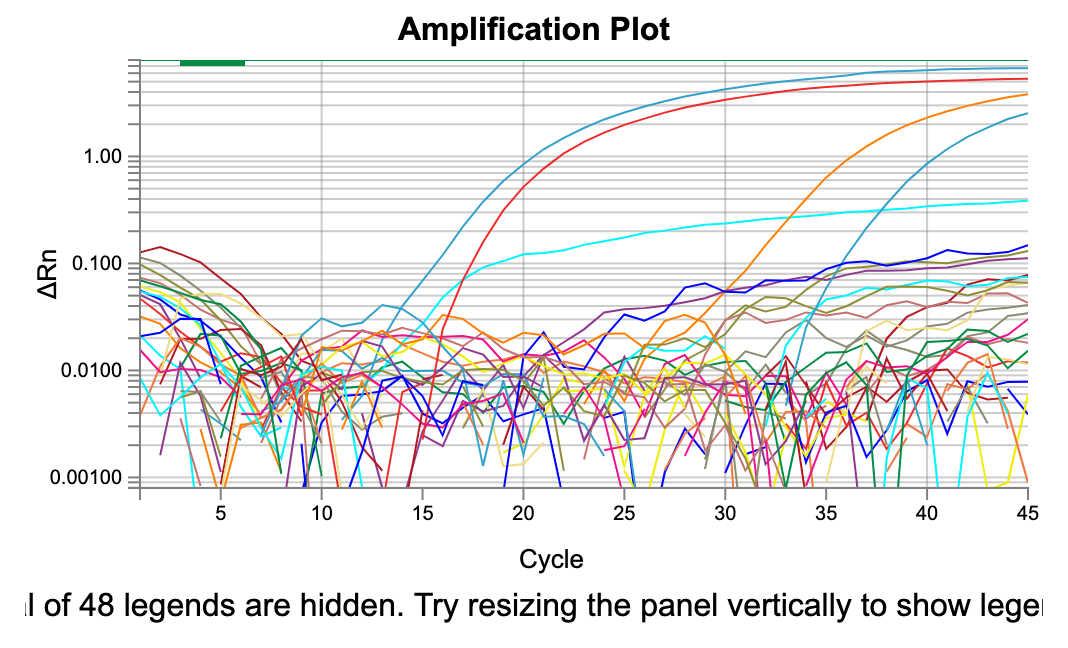

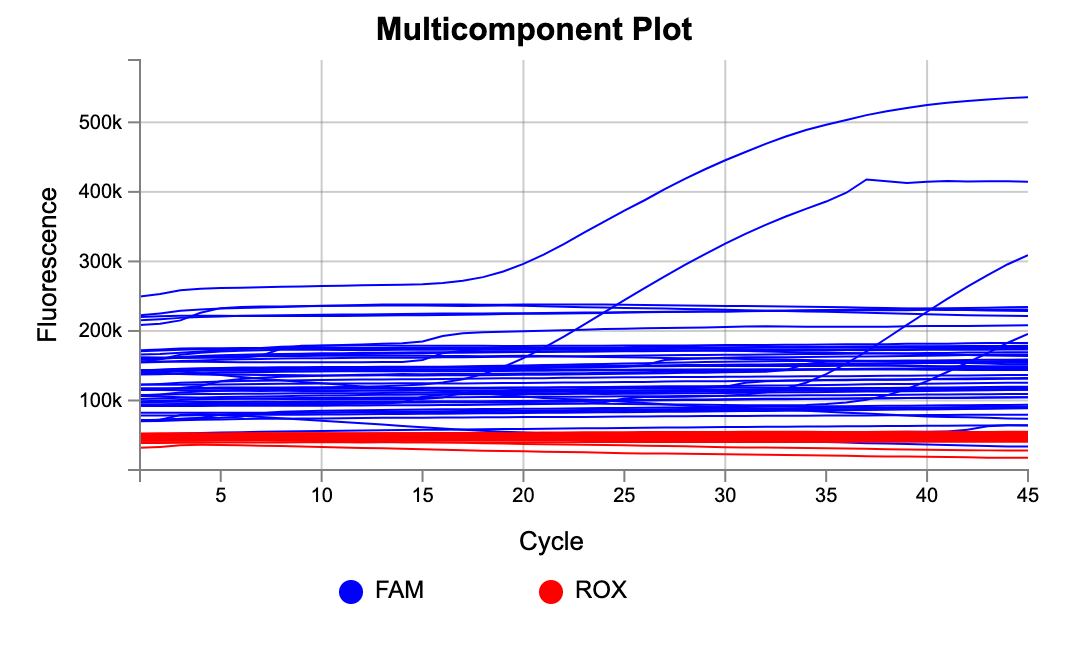

Supplement: Supplement 13 [file media-13.docx]
