# Supplementary material for: Simultaneous detection and quantification of multiple pathogen targets in wastewater
Source: medRxiv. 2023 Dec 5:2023.06.23.23291792. Originally published 2023 Jun 29. Preprint. [Version 2] doi: 10.1101/2023.06.23.23291792 (PMC10327253; doi:10.1101/2023.06.23.23291792)

**S4 Fig.** Skim Milk Flocculation TAC Boxplot Results by gene target

Boxplot for Skim Milk Flocculation – DNeasy PowerSoil Pro Manual extractions (n=30). The dashed line represents the log_10_-transformed theoretical limit of detection (1 gene copy per reaction).


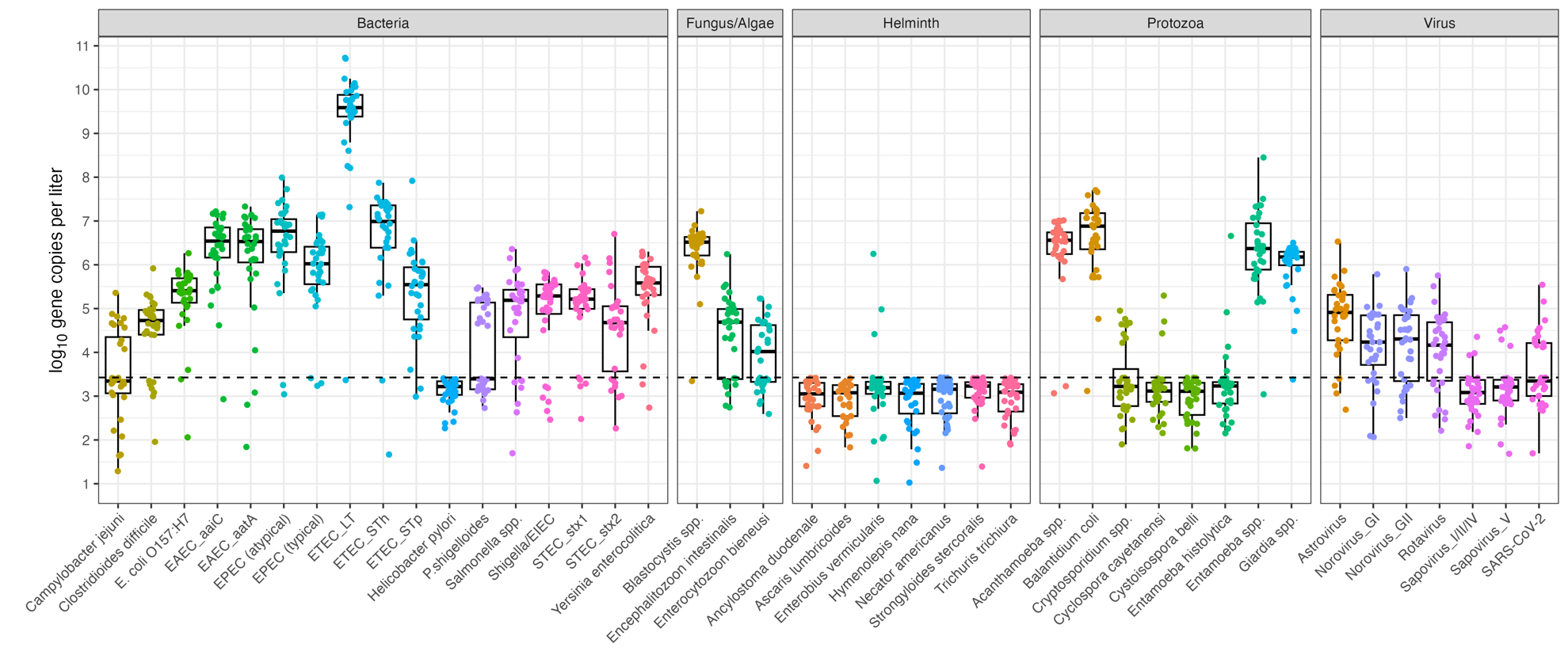

Supplement: Supplement 14 [file media-14.docx]
