# Supplementary material for: Simultaneous detection and quantification of multiple pathogen targets in wastewater
Source: medRxiv. 2023 Dec 5:2023.06.23.23291792. Originally published 2023 Jun 29. Preprint. [Version 2] doi: 10.1101/2023.06.23.23291792 (PMC10327253; doi:10.1101/2023.06.23.23291792)
Supplement: Supplement 16 [file media-16.docx]

**S2 Fig.** InnovaPrep Concentrating Pipette Pellet TAC Results

Boxplot for InnovaPrep Concentrating Pipette Pellet – DNeasy PowerSoil Pro Manual extractions (n= 28 [N=30, 2 duplicates included in plots below]). The dashed line represents the log_10_-transformed theoretical limit of detection (1 gene copy per reaction).

**
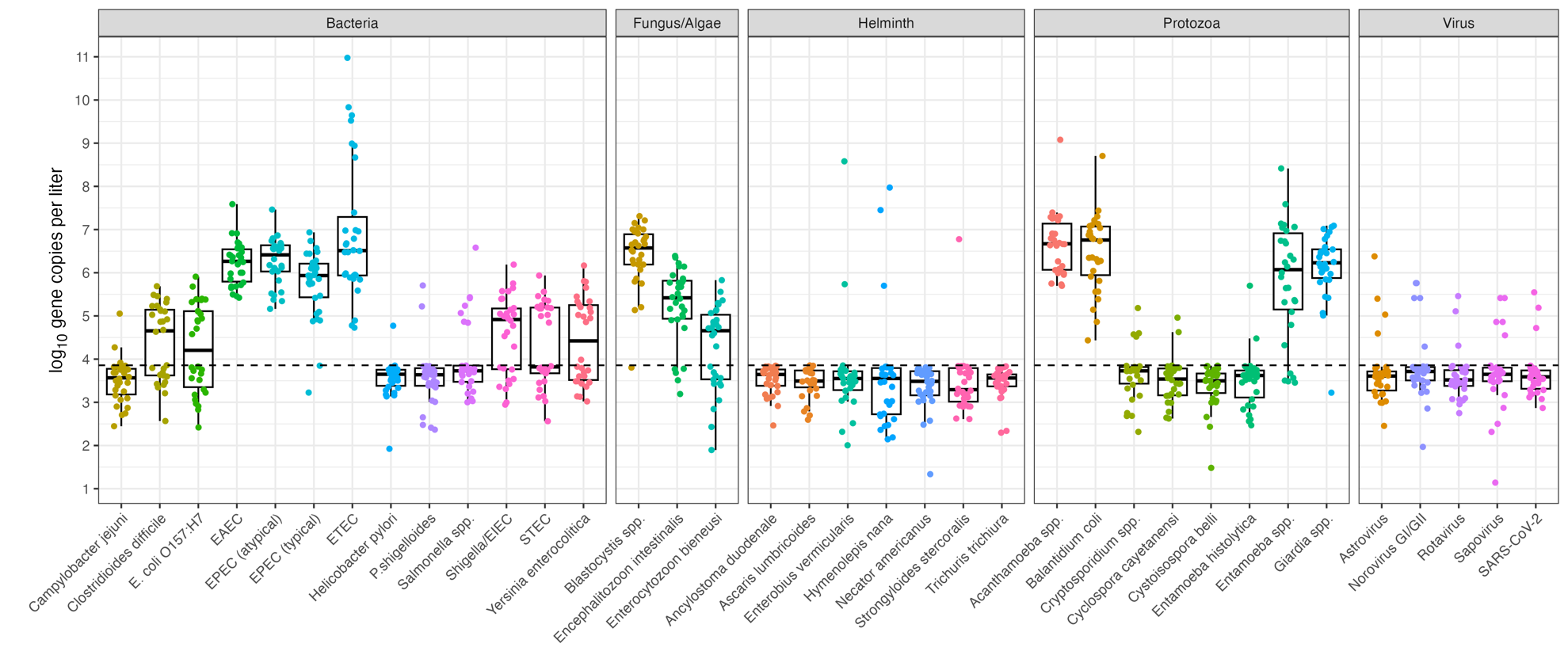
**

We processed an average of 139 mL through the InnovaPrep CP and the average CP pellet was 1.2 mL.
