# Supplementary material for: Simultaneous detection and quantification of multiple pathogen targets in wastewater
Source: medRxiv. 2023 Dec 5:2023.06.23.23291792. Originally published 2023 Jun 29. Preprint. [Version 2] doi: 10.1101/2023.06.23.23291792 (PMC10327253; doi:10.1101/2023.06.23.23291792)

**S1 Fig.** BCoV, PMMoV, mtDNA dPCR RFU plots displaying threshold partitioning for samples, positive and no template controls (NTC)
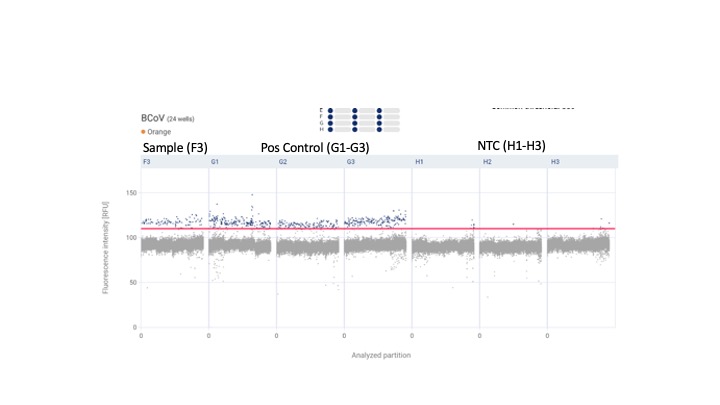

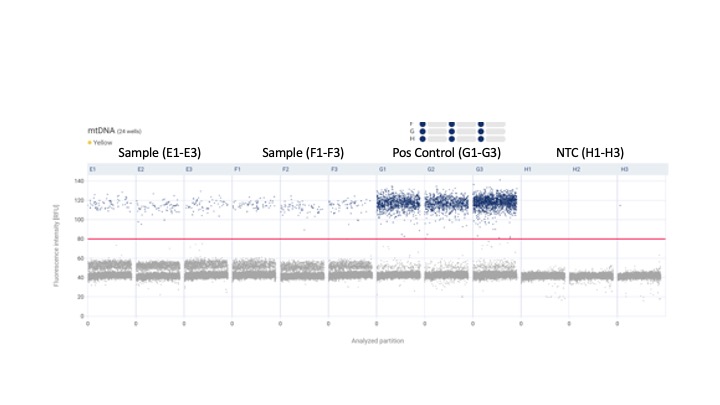

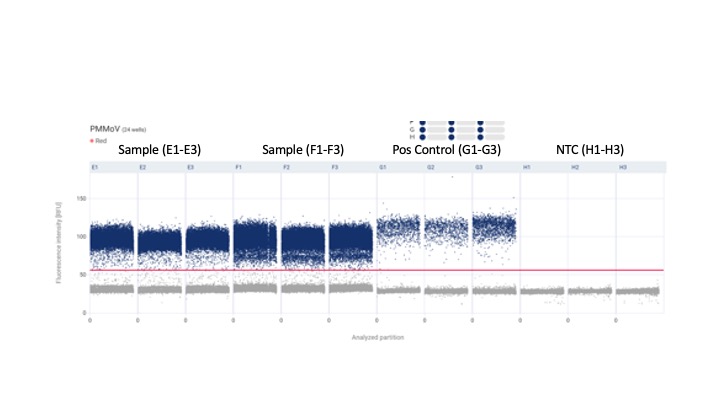

Supplement: Supplement 17 [file media-17.docx]
